# A randomized controlled trial on a self-guided Internet-based intervention for gambling problems

**DOI:** 10.1038/s41598-021-92242-8

**Published:** 2021-06-22

**Authors:** Lara Bücker, Josefine Gehlenborg, Steffen Moritz, Stefan Westermann

**Affiliations:** Department of Psychiatry and Psychotherapy, University Medical Center Hamburg-Eppendorf, Martinistrasse 52, 20246 Hamburg, Germany

**Keywords:** Psychology, Human behaviour

## Abstract

The majority of individuals with problematic and pathological gambling remain untreated, and treatment barriers are high. Internet-based interventions can help to address existing barriers, and first studies suggest their potential for this target group. Within a randomized controlled trial (*N* = 150) with two assessment times (baseline and post-intervention), we aimed to investigate the feasibility, acceptance, and effectiveness of a self-guided Internet-based intervention targeted at gambling problems. We expected a significant reduction in gambling symptoms (primary outcome) and depressive symptoms as well gambling-specific dysfunctional thoughts (secondary outcomes) in the intervention group (IG) compared to a wait-list control group with access to treatment-as-usual (control group, CG) after the intervention period of 8 weeks. Results of the complete cases, per protocol, intention-to-treat (ITT), and frequent user analyses showed significant improvements in both groups for primary and secondary outcomes but no significant between-group differences (ITT primary outcome, *F*(1,147) = .11, *p* = .739, ηp2 < .001). Moderation analyses indicated that individuals in the IG with higher gambling and depressive symptoms, older age, and comorbid anxiety symptoms showed significant improvement relative to the CG. The intervention was positively evaluated (e.g., 96.5% rated the program as useful). Possible reasons for the nonsignificant between-group differences are discussed. Future studies should include follow-up assessments and larger samples to address limitations of the present study.

*Trial Registration* ClinicalTrials.gov (NCT03372226), http://clinicaltrials.gov/ct2/show/NCT03372226, date of registration (13/12/2017).

## Introduction

The majority of individuals with problematic or pathological gambling behavior are not in psychotherapeutic treatment^1^. The estimated treatment gap for this disorder is much larger than for most other psychological disorders, with approximately 90% being untreated^2^. Individual barriers of those affected must be distinguished from institutional barriers. Individuals with gambling problems usually have poor or ambivalent treatment motivation and many either deny their problems, are ashamed of them, or feel that they can manage them on their own^1,3–7^. Although effective treatments for gambling problems exist (e.g., cognitive behavioral therapy (CBT) or motivational interviewing^8–10^), those affected often do not know about these treatments and few psychotherapists specialize in treating gambling problems^11^. Since conventional treatment options are apparently unable to attract individuals with gambling problems, alternative forms of treatment are needed that better address the existing treatment barriers.

Internet-based interventions for psychological disorders (e.g., depression, anxiety) represent a promising alternative to conventional face-to-face treatment as they are able to address specific treatment barriers and maintain effectiveness^12–14^. These interventions can reach individuals who avoid personal contact (e.g., due to feelings of shame or concerns about anonymity), live in rural areas, or have problems with mobility. In addition, they are easily accessible and are available at lower costs compared to conventional treatment^15–17^. Internet-based interventions are either guided (with personal therapeutic support via e-mails, telephone calls, messages), self-guided (without personal therapeutic support), or blended (combination of an Internet-based intervention and classical face-to-face treatment)^18,19^. Such interventions have been intensively researched in recent years for a number of psychological disorders with regard to efficacy, acceptance, adherence, and side effects^13,14,20–23^. In this context, guided interventions have proven to be the most effective in terms of symptom reduction, with medium to large effect sizes in, for example, affective disorders^12,13^. Although smaller effects are found with self-guided interventions, some specific advantages apply as they do not require many resources (e.g., no therapists are needed), are less costly, can be started without a waiting period, and can be used in a completely flexible manner depending on the individual's needs^14^.

Most studies on the effectiveness of Internet-based intervention have been conducted for anxiety disorders and depression^13,20,24^. Whether their results also apply to individuals with gambling problems is still to be seen (to date, no meta-analysis has been published). Several randomized controlled trials (RCTs) found positive effects of Internet-based interventions in the reduction of pathological gambling symptoms^25–27^. One study compared an Internet-based CBT program (iCBT) with therapist contact via e-mail and weekly telephone calls with a wait-list control group and found a large composite between group effect size (Cohen’s *d* = 0.83) for improvements in pathological gambling, anxiety, depression and quality of life at post treatment in favor of the intervention group^25^. Another study investigated the efficacy of iCBT in comparison to an active control group that received monitoring, feedback, and support via the Internet (iMFS) as well to a wait-list control group. Both active conditions resulted in significant reductions in gambling severity; iCBT outperformed iMFS on several other variables (e.g., gambling urges, stress, life satisfaction, and treatment satisfaction^26^). In a prior study, we examined the use of a self-guided iCBT for depression in a sample of individuals with problematic slot machine gambling and compared it with a wait-list control group. We found moderate to strong effect sizes in the reduction of depressive and gambling symptoms favoring the intervention group^27^.

Results regarding the superiority of guided over purely self-guided programs for gambling problems, however, are not that clear. One study compared self-guided iCBT with guided iCBT (e-mail guidance) and found no difference in effectiveness between groups; both led to a significant reduction in gambling symptom severity, urges, frequency, expenditure and psychological distress at 2- and 3-month follow-up, but the guided iCBT also resulted in significant improvements in quality of life and had higher rates of clinically significant changes^28^. Another study investigating different forms of Internet-based interventions in a sample of individuals with gambling problems found no difference in changes of gambling symptom severity of a guided form compared to an unguided one^29^. Moreover, more dropouts were observed in the group receiving the guided intervention relative to the control condition. In addition, a recent study found no difference in symptom reduction between groups of individuals with gambling problems that either received an extended online self-management tools intervention or a brief online normative feedback intervention (personalized feedback report on frequency and problem severity as well as short advice on how to reduce gambling behavior), yet both groups significantly improved over time regarding days gambling and symptom severity^30^.

Given that studies with individuals with gambling problems do not speak for a superiority of guided interventions and considering the high degree of shame and social avoidance, self-guided Internet-based interventions may be more suitable for the treatment of gambling problems. Although guidance may not be beneficial for this target group, the length and intensity of the self-guided intervention is crucial. For example, a meta-analysis found that more intensive self-guided interventions (≥ 6 sessions/modules) were more effective than short or low-intensity interventions for disordered gambling^31^.

Since gambling problems are strongly associated with depression^32^, in a prior study we investigated the efficacy of an Internet-based intervention for depression in a sample of individuals with problematic and pathological slot machine gambling^27^. The self-guided Internet-based intervention deprexis (for a meta-analysis, see^33^) led to a significant reduction in both depressive and gambling-related symptoms compared to a wait-list control group with access to treatment-as-usual (TAU). However, the results of the subjective evaluation showed that the participants felt that their problems were only partially addressed by the program (the program did not deal with gambling-related problems). Thus, we developed a self-guided Internet-based intervention, called Restart, adapted for pathological gambling problems.

In the framework of an RCT with one intervention group that received Restart over 8 weeks and had access to TAU (IG) and one wait-list control group with access to TAU (CG), we investigated the feasibility, acceptance, and effectiveness of the intervention in a sample of individuals with self-reported gambling problems. We expected a significant reduction in problematic gambling behavior (primary outcome) as well as depression- and gambling-specific dysfunctional attitudes and beliefs (secondary outcomes) in the experimental condition. Furthermore, we were interested in variables that moderate treatment outcome. Based on prior findings^27^, we expected that the intervention would be more effective in those with more a more severe gambling problem.

## Results

**Figure 1:**
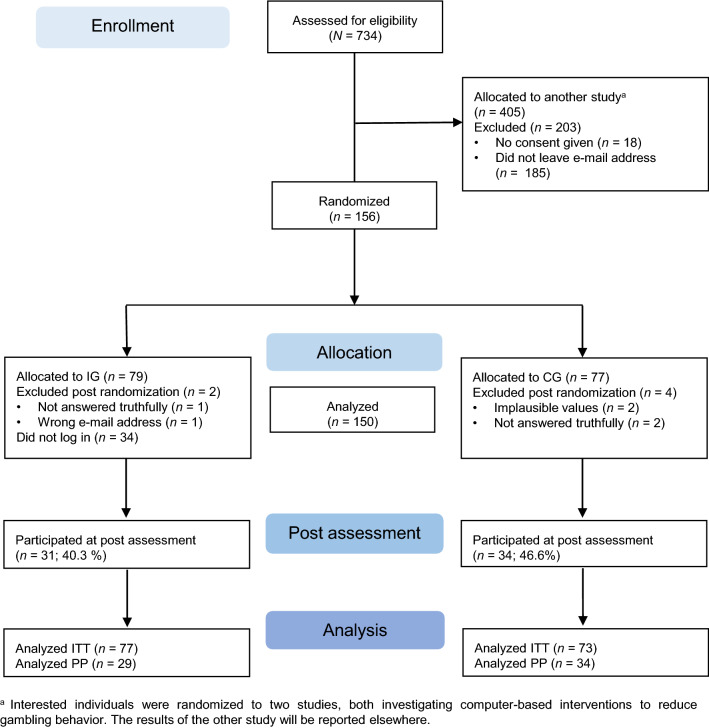
CONSORT flowchart.

A total of 150 participants were included. The study flowchart is depicted in Fig. 1. Baseline characteristics and psychopathology are presented in Table 1. Most participants were male (67.3%) and middle-aged (*M* = 35.03, *SD* = 11.27). Only a small subgroup was currently in psychotherapy (11.3%), was using self-help (4.0%) or was taking psychotropic medication (8.0%) at the beginning of the study participation. Overall, the sample reported moderate symptoms within the baseline assessment (Patient Health Questionnaire Depression Module-9, PHQ-9: *M* = 11.08, *SD* = 4.69; Pathological Gambling Adaptation of Yale-Brown Obsessive Compulsive Scale, PG-YBOCS total: *M* = 19.24, *SD* = 6.22; Gambling Attitudes and Beliefs Scale, GABS: *M* = 19.87, *SD* = 8.62; South Oaks Gambling Screen, SOGS: *M* = 10.34, *SD* = 2.91). The following information on lifetime diagnoses was reported by the total sample: 20.7% gambling disorder, 0.7% obsessive–compulsive disorder, 8.0% anxiety disorder, 18.0% depression, 5.3% posttraumatic stress disorder, and 6.0% alcohol/drug dependency. There were no significant differences in any of the baseline characteristics between the two groups.

**Table 1:** Baseline characteristics (intention-to-treat sample), frequency, means, and standard deviation (in brackets).

	IG (*n* = 77)	CG (*n* = 73)
**Demographic characteristics**		
Gender (% male)	64.9	69.9
Age in years	33.83 (11.26)	36.29 (11.22)
Completed high school (%)	18.2	23.3
Nationality (% German)	81.8	84.9
Professional status (% employed full-time)	64.9	64.4
**Treatment variables**		
Currently in psychotherapy (%)	10.4	12.3
Currently taking psychotropic medication (%)	7.8	8.2
Currently using self-help (%)	2.6	5.5
Age at first gambling	19.66 (15.81)	21.51 (10.23)
Age at frequent gambling	22.90 (16.63)	21.66 (22.80)
Currently in gambling suspension (%)	11.7	19.2
**Psychopathology**		
PHQ-9^a^	10.80 (4.34)	11.35 (5.02)
GABS^b^	19.80 (9.01)	19.95 (8.30)
SOGS^c^ total score	10.18 (2.85)	10.49 (3.00)
PG-YBOCS^d^ total score	18.69 (5.94)	19.77 (6.47)
PG-YBOCS^d^ behavior	9.03 (3.21)	9.91 (3.31)
PG-YBOCS^d^ thoughts	9.66 (3.14)	9.86 (3.53)
Gambling disorder (%)	19.5	21.9
Obsessive–compulsive disorder (%)	1.3	0
Anxiety disorder (%)	9.1	6.8
Depression (%)	20.8	15.1
Posttraumatic stress disorder (%)	3.9	6.8
Alcohol/drug dependency (%)	2.6	9.6

^a^PHQ-9: Patient Health Questionnaire–9.

^b^Gambling Attitudes and Beliefs Survey.

^c^South Oaks Gambling Screen.

^d^Pathological Gambling Adaptation of Yale-Brown Obsessive Compulsive Scale.

Post-intervention, 10.8% of the total sample was currently in treatment (IG 9.7%, CG 11.8%), 7.7% was using some kind of self-help (IG 6.5%, CG 8.8%), and 10.8% was taking medication (IG 16.1%, CG 5.9%). There were no significant differences between the two groups regarding these variables (all *p* < .05).

### Primary outcome

The results of the paired sample *t*-tests for within-group differences are presented in Table 2, and the results of the ANCOVA analyses for complete cases (CC), per protocol (PP), intention-to-treat (ITT), and frequent users are summarized in Table 3. Missing values in the ITT analyses were replaced using two methods—expectation maximization (EM) and multiple imputation (MI)—to take into account that there is no gold standard method for estimating or replacing missing values. Regarding the primary outcome (reduction in pathological gambling measured with the PG-YBOCS), no significant between-group differences was observed for all samples. A significant improvement for the primary outcome was observed for the complete cases of both groups, with a strong effect size for the IG (*t*(30) = 5.05, *p* < .001, Cohen’s *d* = − 1.17) and a medium effect size for the CG (*t*(33) = 3.50, *p* = .001, Cohen’s *d* = − 0.72).

**Table 2:** Within-group differences across time of complete cases with means, standard deviations, effect sizes in Cohen’s *d* and 95% confidence intervals.

Measurements	IG (*n* = 31)			CG (*n* = 34)		
	Pre	Post	Cohen’s *d* [95% CI]	Pre	Post	Cohen’s *d* [95% CI]
PG-YBOCS^a^ total	20.77 (6.96)	13.29 (5.72) [****]	− 1.17 [− 1.94 to − 0.41]	19.21 (5.23)	14.33 (7.98) [****]	− 0.72 [− 1.42 to − 0.03]
PHQ-9^b^	12.07 (5.82)	9.68 (5.19) [*]	− 0.43 [− 1.15 to 0.28]	11.91 (3.71)	10.53 (5.29)	− 0.30 [− 0.98 to 0.37]
SOGS^c^	10.61 (3.06)	6.74 (3.88) [****]	− 1.11 [− 1.87 to − 0.35]	10.27 (2.99)	6.59 (3.29) [****]	− 1.17 [− 1.90 to − 0.44]
PG-YBOCS^a^ behaviour	10.52 (3.62)	6.32 (3.53) [****]	− 1.18 [− 1.94 to − 0.41]	9.50 (2.48)	6.97 (4.19) [****]	− 0.74 [− 1.43 to − 0.04]
PG-YBOCS^a^ thoughts	10.26 (3.73)	6.97 (2.89) [****]	− 0.99 [− 1.73 to − 0.24]	9.71 (2.96)	7.35 (3.93) [***]	− 0.98 [− 1.37 to 0.01]
GABS^d^	21.61 (9.18)	18.36 (11.26)	− 0.32 [− 1.03 to 0.39]	20.62 (8.10)	16.82 (9.74) [****]	− 0.42 [− 1.10 to 0.26]

[*] = *p* ≤ .05; [**] = *p* ≤ .01; [***] = *p* ≤ .005; [****] = *p* ≤ .001.

^a^ Pathological Gambling Adaptation of Yale-Brown Obsessive Compulsive Scale.

^b^ PHQ-9: Patient Health Questionnaire–9.

^c^ South Oaks Gambling Screen.

^d^ Gambling Attitudes and Beliefs Survey.

**Table 3:** Between-group difference pre-post; ANCOVAs with baseline scores as covariates.

Measurements	Complete cases (*n* = 65)	Per protocol (*n* = 63)	Frequent user (*n* = 47)	Intention-to-treat (*n* = 150)*
PG-YBOCS^a^ total	*F*(1,62) = .70, *p* = .408, η_p_^2^= .011	*F*(1,60) = .90, *p* = .346, η_p_^2^ = .015	*F*(1,44) = .03, *p* = .868, η_p_^2^ = .001	*F*(1,147) = .11, *p* = .739, η_p_^2^ < .001, [*p* = .448]
PHQ-9^b^	*F*(1,62) = .56, *p* = .456, η_p_^2^ = .009	*F*(1,60) = 1.38, *p* = .245, η_p_^2^ = .022	*F*(1,44) = .45, *p* = .505, η_p_^2^ = .010	*F*(1,147) = .13, *p* = .722, η_p_^2^ = .001, [*p* = .769]
SOGS ^c^	*F*(1,62) = .00, *p* = .970, η_p_^2^ < .001	*F*(1,60) = .34, *p* = .562, η_p_^2 ^= .006	*F*(1,44) = .29, *p* = .592, η_p_^2^ = .007	*F*(1,147) = .01, *p* = .921, η_p_^2^ < .001, [*p* = .702]
PG-YBOCS^a^ behaviour	*F*(1,62) = 1.03, *p* = .315, η_p_^2^ = .016	*F*(1,60) = 1.52, *p* = .223, η_p_^2 ^= .025	*F*(1,44) = .04,* p* = .835, η_p_^2^ = .001	*F*(1,147) = .66, *p* = .418, η_p_^2^ = .004, [*p* = .444]
PG-YBOCS^a^ thoughts	*F*(1,62) = .30, *p* = .588, η_p_^2^ = .005	*F*(1,60) = .27, *p* = .608, η_p_^2^ = .004	*F*(1,44) = .003,* p* = .958, η_p_^2^ < .001	*F*(1,147) = .02, *p* = .900, η_p_^2^ < .001, [*p* = .556]
GABS^d^	*F*(1,62) = .13, *p* = .717, η_p_^2^ = .002	*F*(1,60) = .09, *p* = .764, η_p_^2^ = .002	*F*(1,44) = .17,* p* = .682, η_p_^2^ = .004	*F*(1,147) = .04, *p* = .851, η_p_^2^ < .001, [*p* = .798]

* Intention-to-treat analyses were computed with expectation maximization as the method for replacing missing values. The square brackets contain the *p*-value of analyses with multiple imputation as the method for replacing missing values.

^a^ Pathological Gambling Adaptation of Yale-Brown Obsessive Compulsive Scale.

^b^ PHQ-9: Patient Health Questionnaire–9.

^c^ South Oaks Gambling Screen.

^d^ Gambling Attitudes and Beliefs Survey.

### Secondary outcomes

No significant group differences emerged for any of the CC, PP, ITT, or frequent user analyses on the secondary outcomes (all *p* > .05). Paired sample *t*-tests of complete cases showed a significant reduction in depression (PHQ-9), with a small effect size for the IG (*t*(30) = 2.13, *p* = .041, Cohen’s *d* = − 0.43) but not for the CG (*t*(33) = 1.58, *p* = .123, Cohen’s *d* = − 0.30). For the reduction in pathological gambling measured with the SOGS, within-group differences were strongly significant for both groups (IG *t*(30) = 5.92, *p* < .001, Cohen’s *d* = − 1.11; CG *t*(33) = 6.32, *p* < .001, Cohen’s *d* = − 1.17). Pairwise comparisons for the reduction of gambling-specific cognitive distortions (GABS) were significant for the CG (*t*(33) = 3.88, *p* < .001, Cohen’s *d* = − 0.42) and failed to reach a conventional level of significance for the IG (*t*(30) = 1.98, *p* = .057, Cohen’s *d* = − 0.32).

### Feasibility

#### Completion and usage

A total of 65 (43.3%) participants completed the post assessment. Completers and non-completers differed on several baseline characteristics. More women were among the completers (41.5%; 27/65) than among the dropouts (25.9%; 22/85; χ^2^ (1,150) = 4.11, *p* = .043) and completers more often received a diagnosis of depression (26.2%; 17/65) relative to non-completers (11.8%; 10/85; χ^2^ (1,150) = 5.17, *p* = .023). Completers had used self-help materials more often in the past (15.4%; 10/65) than non-completers (3.5%; 3/85; χ^2^ (1,150) = 6.54, *p* = .011). Completers were relatively older (*M* = 37.29, *SD* = 11.41) compared to non-completers (*M* = 33.29, *SD* = 10.91; *t*(148) = − 2.18, *p* = .031) and had higher baseline depressive symptomatology (PHQ-9: *M* = 11.99, *SD* = 4.78) compared to non-completers (PHQ-9: *M* = 10.39, *SD* = 4.52; *t*(148) = − 2.09, *p* = .038). Regarding all other baseline variables, completers and non-completers did not differ.

The majority (68.8%, 53/77) of the IG logged into the program at least once. Of those, 33.9% (18/53) completed 1 or 2 modules, 13.2% (7/53) completed 3 or 4 modules and 9.5% (5/53) completed 7 or more modules. The average time of usage over the 8-week intervention period was 79.66 min (*SD* = 105.51), the mean number of completed modules was 1.74 (*SD* = 2.69).

#### Subjective appraisal

The subjective appraisal ratings of Restart are depicted in Tables 4 and 5. Most participants evaluated the intervention positively and indicated their perceived benefit. Of the users who provided information on their subjective evaluation, 96.5% indicated that they considered the program suitable for self-application, found that the instructions were understandable, and that the program was useful. However, 37.9% indicated that the program was not relevant for their gambling-related symptoms, and 74.4% indicated that they had to push themselves to use the program.

**Table 4:** Subjective appraisal of Restart (*n* = 29).

Items	*M (SD)*	% positive
1. I think the program is suitable for self-application	3.07 (0.80)	96.5
2. My gambling problem was reduced due to my using the program	2.69 (1.00)	89.6
3. I think the instructions were written understandably	3.24 (0.79)	96.5
4. I think the program was useful	3.04 (0.73)	96.5
5. I was able to use the program regularly over the past several weeks	2.28 (0.92)	82.7
6. I had to push myself to use the program	2.31 (1.04)	74.4
7. I consider the program to be a useful adjunct to psychotherapy	2.83 (0.89)	89.6
8. The program is not relevant to my gambling-related symptoms	1.52 (0.75)	37.9

Answers were given on a 4-point rating scale; 1 = not true at all, 2 = somewhat true, 3 = mostly true, 4 = completely true.

**Table 5:** Subjective appraisal of Restart based on the German version (ZUF-8) of the Client Satisfaction Questionnaire (CSQ-8; *n* = 25).

Items	*M (SD)*	% positive
How do you rate the quality of the program? (Excellent, good, okay, not good)^a^	1.96 (0.84)	88.0
Did you receive the type of treatment you expected to receive? (Absolutely, a lot, a little, not at all)	3.40 (1.04)	68.0
To what extent did the program help you cope with your problems? (Absolutely, a lot, a little, not at all)^a^	2.40 (1.12)	72.0
Would you recommend the program to a friend with similar symptoms? (Yes, probably yes, probably not, no)	3.32 (0.85)	84.0
How happy are you with the extent of the help you have received through using the program? (Very satisfied, mostly satisfied, somewhat dissatisfied, dissatisfied)	3.20 (1.08)	68.0
Did the program help you to cope with your problems more successfully? (Absolutely, a lot, a little, not at all)^a^	2.36 (1.35)	74.0
How satisfied are you with the program in general? (Very satisfied, mostly satisfied, somewhat unsatisfied, unsatisfied)^a^	2.36 (1.32)	72.0
Would you use the program again? (Yes, probably yes, probably not, no)	3.52 (0.96)	76.0

^a^ A lower score indicates more positive answers (inverted scores).

### Moderation analysis

Significant interactions of the exploratory moderation analysis of the ITT sample are presented in Table 6. Positive betas indicate that higher scores on the moderator led to a greater reduction on the primary outcome (PG-YBOCS total difference from pre to post) relative to the control group. The last three columns specify the difference between both groups with regard to baseline to post change scores at different levels of the moderator (low = *p* for − 1 *SD*; average = *p* for 0; high = *p* for + 1 *SD*). Individuals in the IG who were older, who indicated a diagnosis of pathological gambling, who indicated to have a diagnosis of a mental disorder, or who affirmed comorbid anxiety symptoms showed significant improvement relative to the CG. Furthermore, those individuals in the IG who scored higher on baseline gambling and depression symptoms (SOGS and PHQ-9) and those, who were currently not using other self-help, benefited more compared to the CG. Lastly, satisfaction with the program positively influenced the treatment effect compared to the CG.

**Table 6:** Moderators for problem gambling improvement (Pathological Gambling Adaptation of Yale-Brown Obsessive Compulsive Scale–total difference scores, means are centered); results of intention-to-treat sample (*N* = 150).

Outcome Parameter	*B*	*SE*	*t*	*p*	LLCI	ULCI	*p* for –1SD	*p* for 0	*p* for + 1SD
Age	0.201	0.099	2.045	.043	0.007	0.396	.530	.492	.018
Gambling disorder diagnosis	6.944	2.736	2.548	.012	1.538	12.351	.826	–	.007
PTBS^a^ diagnosis	12.216	5.026	2.431	.016	2.283	22.148	.551	–	.009
No diagnosis	− 6.042	2.258	− 2.677	.008	− 10.504	− 1.581	.008	–	.361
Current self-help	− 14.806	5.958	− 2.485	.014	− 26.582	− 3.030	.116	–	.027
WSQ^b^ item 3 (general anxiety disorder)	4.250	1.307	1.307	.001	1.666	6.834	.510	.510	.009
WSQ^b^ item 5 (agoraphobia)	− 5.6217	2.817	− 1.995	.048	− 11,190	− 0.054	.029	–	.980
WSQ^b^ item 6 (specific phobia)	− 7.625	2.755	− 2.68	.006	− 13.069	− 2.181	.003	–	.801
WSQ^b^ item 7 (specific phobia)	− 5.908	2.315	− 2.552	.012	− 10.482	− 1.333	.008	–	.550
WSQ^b^ item 8 (social phobia)	− 5.219	2.327	− 2.243	.026	− 9.818	− 0.619	.018	–	.587
Gambling due to feelings of luck	− 6.482	2.202	− 2.945	.004	− 10.833	− 2.131	.008	–	.125
PHQ-9^c^ total scale	0.558	0.228	2.448	.016	0.108	1.009	.211	.428	.029
SOGS^d^ total Scale	0.904	0.382	2.368	.019	0.150	1.658	.244	.151	.023

*B* = beta coefficient, *SE* = standard error; LLCI = lower limit confidence interval; ULCI = upper limit confidence interval; ^a^ PTBS = post-traumatic stress disorder; ^b^ WSQ = Web Screening Questionnaire; ^c^ PHQ-9 = Patient Health Questionnaire–9; ^d^ SOGS = South Oaks Gambling Screen. The last three columns present the *p*-values when the values are one standard deviation below, above, and equal to the mean.

## Discussion

In this randomized controlled trial, we investigated the feasibility, acceptance and effectiveness of a new self-guided Internet-based intervention for problematic gambling. Although the intervention group (i.e., those participants who had access to Restart during the study intervention period of 8 weeks) improved in terms of the primary and almost all secondary outcomes (except for gambling specific dysfunctional attitudes and beliefs, GABS), no significant group differences could be observed (neither for the CC, PP, the frequent user nor the ITT analyses). On first sight, this finding is inconsistent with previous RCTs investigating the effectiveness of Internet-based interventions for pathological gambling^25–27,29^. Surprisingly, the CG (with access to TAU) improved to almost the same extent as the intervention group. This is particularly surprising in light of our previous study demonstrating the effectiveness of a depression-focused Internet-based intervention in a sample of individuals with slot machine gambling problems^27^. In the current study, we had expected greater effectiveness because of the program's focus on gambling-related topics but instead found no effects. This may be attributable to the characteristics of the sample. For example, the first study only included individuals who engaged in slot machine gambling, whereas the current study did not differentiate between different types of gambling. In addition, the inclusion criteria of the first study defined that a depressive symptomatology had to be present as well as a gambling problem. This may have had an effect as we found in the present study that individuals with initially more pronounced depressive symptoms benefited significantly more from the intervention. However, we found that depressive symptoms at baseline were very similar in both samples and even slightly higher in the second study (PHQ-9: 10.9% vs. 11.8%), which means that the reasons are probably to be found elsewhere. In the following paragraphs, we offer several possible reasons that may explain the absence of between-group effects.

One reason could be spontaneous recovery in the control group. Slutske and colleagues reported a recovery rate of 40% in pathological gambling; of those, 82% recovered without treatment (a phenomenon that is called ‘natural recovery’ or ‘spontaneous remission’) suggesting that this is not uncommon in pathological gambling^2^. However, there are some important points to note in this regard, as ‘recovery’ is often defined differently^34^. If, for example, only the number of gambling activities in the past year is considered, any remaining gambling symptoms are ignored. It is also unclear how long an individual must be symptom-free to be considered recovered. Furthermore, individuals who are currently not experiencing addictive behavior often report that they no longer suffer from symptoms, but they still relapse as the course of pathological gambling is often fluctuating^35^. This might be relevant for the interpretation of our results as it is conceivable that individuals who decided to seek help and to participate in the study during an acute phase of gambling (in which symptoms such as chasing, lying, strong gambling impulses and frequent thoughts on gambling are present) will not experience those symptoms at a later time (when the acute phase has passed), for example at the post assessment 8 weeks following the baseline assessment. In order to be able to assess such effects, future studies should plan longer follow-up assessments. Also, at the post assessment, a similar number of study participants were currently being treated as at the baseline assessment (baseline 11.3% vs. post 10.8%). Here, slightly more participants in the control group were in treatment (CG 11.8% vs. IG 9.7%) and likewise, slightly more participants in the control group were using self-help at post assessment (CG 8.8% vs. IG 6.5%). With regard to medication, however, there was a higher use in the intervention group (IG 16.1% vs. CG 5.9%). None of these differences were significant, though, so it cannot be assumed that these aspects had an influence on the efficacy results.

Another possible reason for the improvement in the two conditions might be that solely the first initiative or the decision to seek help led to a sustained reduction of symptoms in both groups or, it might result from a self-selection bias (i.e., decision to participate in the study may be correlated with several personality traits or demographic characteristics making the sample unrepresentative)^36^. However, a study investigating the self-selection bias in an Internet treatment trial for depression found that very few variables were independently associated with study participation and none of these were associated with either the primary outcome nor any of the secondary treatment outcomes^37^. Another explanation for the improvement of both the intervention group as well as the wait-list control group could be regression to the mean (i.e., the second measure following an extreme measure is closer to the mean)^38^.

Yet another reason could be the choice of control group: although scientists consistently worry about the use of wait-list control groups in clinical trials because they might result in an overestimation of treatment effects^39–41^, several studies found improvements in wait-list control groups^27,42–44^, which could stem from participants having the prospect of being able to use the program afterwards. This, in turn, might instill them with hope and therefore lead to relief and a reduction of symptoms. Although our control group also had access to TAU it must rather be considered as a wait-list control group since the use of TAU was available to the whole sample (i.e., also to the intervention group).

It is also conceivable that the intervention itself has some shortcomings that led to the lack of effects. For example, it could be that the texts or the individual modules were too long. It is also possible that it would have been more effective if the modules had been activated one after the other (although we did not do this because we wanted to ensure that the individual modules did not build on each other in terms of content, this could possibly arouse curiosity). We assume that it makes sense to present users of an Internet-based intervention with smaller units and not to overwhelm them with too many options.

Lastly, the hypothesis that self-guided Internet-based interventions are more appropriate and as effective as guided interventions may be incorrect. For instance, a meta-analysis reported the superiority of guided over unguided programs in individuals with a drinking problem, although older meta-analyses indicated that there was no difference between therapist-assisted bibliotherapy and unguided bibliotherapy in this target group^45^.

We found that several baseline characteristics moderate treatment outcome (reduction of pathological gambling). Individuals of the experimental group at older age, with diagnosed pathological gambling or comorbidities and those with no competing treatments showed significant improvement in gambling symptoms (PG-YBOCS) relative to the control group. Furthermore, those individuals of the intervention group who gamble due to feelings of luck significantly improved more relative to the controls. These results partly correspond to previous study findings, which found a significant interaction between baseline depression severity and treatment outcome indicating that higher symptoms were associated with better outcome in studies on low intensity or Internet-based interventions for depression^46–48^. However, Karyotaki and colleagues^14^ observed that participants improved after using a self-guided Internet-based intervention regardless of their baseline symptom severity. In line with the present findings, in our pilot study on the effectiveness of a self-guided Internet-based intervention for depression in a sample of individuals with gambling problems, we found that the intervention was particularly beneficial for those with high baseline gambling symptoms^27^. A meta-analysis on problem drinking concluded that Internet-based interventions are more beneficial for older adults and men as well as for less educated participants^49^, which partially corresponds to our results. Furthermore, it was found that the treatment effect of a transdiagnostic Internet-based intervention was more pronounced among participants with anxiety disorders (vs. mood disorders)^50^. The finding that the current use of additional self-help moderated treatment outcome could be reconciled by the fact that several different approaches may result in that both interventions are carried out half-heartedly.

The study has several strength and limitations. An aspect that can be either regarded a strength or a weakness is that no diagnosis of a gambling disorder or a specific symptom severity cut-off was firmly required for study participation. Thus, we faced a wide range of symptom severity and therefore an increased risk of a type I error (possibly leading to an underestimation of the true effectiveness of the intervention). In view of the results of the moderation analyses, we assume that the intervention might be effective in a sample of more severely affected individuals. The advantage of our broad recruitment strategy is that more individuals with the (subjective) need for treatment could be included in the study and were allowed access to the intervention. A limitation of the study is the sample size, which was based on a power calculation assuming a medium effect size. A recent meta-analysis, however, only found a small effect size for self-guided Internet-based interventions (transdiagnostic evaluation)^14^, so we need larger samples to detect such small effects. Furthermore, we had to deal with a large number of non-completers as only 43% completed the post assessment and replacement of missing values always results in a reduced statistical power. Other studies on Internet-based interventions for pathological gambling report a wide range of completion rates, ranging from 17 to 90%^25–30^, with several below 50%^26,28,29^. Accordingly, our completion rate can be considered comparable to similar studies. Similar dropout rates are found in studies examining Internet-based interventions for problem drinking^49^. In addition, no follow-up assessments were conducted so that it is not possible to draw conclusions about long term effects (e.g., “sleeper effects”)^51^ or future rates of relapses.

In our study, we were not able to demonstrate the effectiveness of the Internet-based intervention Restart in reducing gambling specific and depressive symptoms. Although the intervention group numerically improved more than the wait-list control group with access to TAU, this group difference was not significant. Moderation analyses suggest greater efficacy in participants with more severe symptomatology; those with higher gambling symptoms and depression as well as older participants and those with comorbid anxiety symptoms benefited to a greater extent from the intervention. This has important clinical implications and should be considered when selecting patients for these interventions. Future studies should include larger samples and also examine long-term effects. In addition, the interventions themselves should be shorter and the content should be delivered in an engaging manner.

## Materials and methods

### Study design

A randomized controlled trial with parallel allocation (1:1) to two conditions was set up. The experimental group received immediate access to an Internet-based self-help intervention (Restart) for an 8-week period, and both groups had access to TAU (i.e., treatments such as outpatient psychotherapy or medication allowed to be continued or used simultaneously); the wait-list control group received full access to Restart after completion of the post assessment. Before and after the intervention period two anonymous online assessments were carried out. The baseline assessment screened for sociodemographic and psychopathological data and confirmed inclusion and exclusion criteria. At post, psychopathological data and subjective appraisal of Restart (only for the intervention group) were assessed. At both assessment points, data was obtained via an Internet survey using ESF Survey (Unipark). After completing the post assessment, participants received a monetary incentive (20 Euros Amazon voucher) and access to another Internet-based intervention that aims at reducing the urge to gamble (approach bias modification for gambling). No personal data (e.g., name, telephone number, address), except for an anonymous e-mail address (instructions for creating such an e-mail address were granted), was requested in any of the surveys. E-mail addresses were automatically recoded and stored non-electronically in a safe while all other data was stored on password-protected computers.

### Ethical statements

The study was approved by the ethics committee of the German Psychological Society (DGPs; ID: SM 092017_amd_012014_2b) and was undertaken in accordance with the Declaration of Helsinki. The authors assert that all procedures contributing to this work comply with the ethical standards of relevant national and institutional guidelines. All participants gave online informed consent before participating in the study. The trial was pre-registered with ClinicalTrials.gov (registration number: NCT03372226; date of registration 13/12/2017) and a study protocol was published at the beginning of the study^52^.

### Participants

All data was collected, stored, and analyzed at the University Medical Center Hamburg-Eppendorf (Germany). Recruitment started in January 2018, the first participant was included on January 20, the last participant enrolled on October 22, 2018. To aid recruitment a Google AdWords campaign in Germany, Switzerland (German-speaking area) and Austria was conducted. The advertisement appeared when relevant words were entered in the Google search engine (e.g., “self-help + gambling” or “treatment + gambling disorder”) and directed to a website providing study information and a link to the baseline survey.

Participants had to fulfill the following criteria to qualify for inclusion: (a) age between 18 and 75 years, (b) confirmation to participate in the study by electronic informed consent, (c) Internet access, (d) sufficient command of the German language, (e) willingness to take part in two online assessments, (f) willingness to use Restart over an 8-week period, (g) willingness to provide an anonymous e-mail address, and (h) subjective experience of psychological and emotional distress and gambling problems, and desire for treatment for gambling-related symptoms. Participants with a lifetime diagnosis of a schizophrenia spectrum disorder or a bipolar disorder were screened out in order to exclude individuals with possible acute delusions whose responses might have limited validity. Additionally, individuals who reported acute suicidality (assessed with one item of the Patient Health Questionnaire-9 depression module, PHQ-9) were excluded from the study. In case of acute suicidal tendencies, facilities and telephone numbers for further help were provided.

### Sample size

Using G*Power^53^ a sample size of 128 (64 per group) was recommended to test for a medium effect of *f* = 0.25 (*α* = 0.05, *β* = 0.80) using analysis of covariance (ANCOVA). Considering an anticipated dropout rate of 20%, we aimed recruiting a sample size of 154 participants (77 per group).

### Randomization

Participants were randomly assigned to the intervention group or the wait-list control group (allocation rule 1:1) by means of a randomization plan. The randomization plan was created with the software Research Randomizer (www.randomizer.org) using block randomization. Because of the online design of this study, no concealment was necessary. Participants started the baseline assessment by following the link on the study website. The first author was informed about each participant’s completion of the baseline assessment via e-mail and then chronologically (based on the finishing time of the online baseline assessment) allocated the participant to one of the two conditions using the randomization plan (the procedure is best described as centralized assignment). There was no personal contact between the first author and the participant before allocation. Participants in the intervention group received an e-mail containing a code and a password for access to the program. Participants in the wait-list control group were informed via e-mail that they would get access to the program after completion of the post assessment 8 weeks later.

### Intervention

The Internet-based self-help intervention Restart consists of 11 modules (see^52^ for a detailed description) addressing gambling-related problems (e.g., handling of gambling impulse, relapse prevention) as well as comorbid emotional symptoms (e.g., low self-esteem, depressive symptoms) that are associated with problematic and pathological gambling^54,55^. The program conveys cognitive-behavioral strategies as well as mindfulness-based and metacognitive elements. The contents of the individual modules are mainly communicated in text format, but the program also includes video and audio clips. Furthermore, all modules are interactive; they incorporate exercises, worksheets, and audio files. Participants were recommended to work on one to two modules per week (30 to 60 min per module) and were free to choose the order of the modules. Restart is a self-guided Internet-based intervention providing the option to contact a personal moderator in case of questions or technical problems with the program; no additional therapeutic support was offered. Via program log files, the length of time the participants were logged into the program could be checked. If an individual participant did not use the program at all, he or she was reminded via e-mail to start the intervention. An initial reminder e-mail was sent 1 week after the baseline survey. For those who did not log in until the post survey, reminder e-mails then followed at 2-week intervals for a total of four e-mails. Of course, no more e-mails were sent once participants had registered. In the study protocol^52^, it was originally planned to incorporate a smartphone app that could be used in combination with the online program. Unfortunately, this was not possible because the app was not fully developed at the start of the study.

### Primary measure

#### The Pathological Gambling Adaptation of Yale-Brown Obsessive Compulsive Scale (PG-YBOCS)

The PG-YBOCS^56^ served as the primary outcome. It assesses past-week gambling severity on two subscales: (1) thoughts about gambling and urge to gamble and (2) gambling behavior. The sum score of the PG-YBOCS ranges from 0 to 40 (20 for each subscale), differentiating between subclinical (0–7), mild (8–15), moderate (16–23), severe (24–31), and extreme (32–40) symptom severity. Internal consistency is good with a Cronbach’s α > 0.90^56^.

### Secondary measures

#### Patient Health Questionnaire-9 Depression Module (PHQ-9)

The PHQ-9^57^ measures depression symptoms over the past 2 weeks on a scale from 0 to 27, with higher scores indicating higher symptom severity. It distinguishes between minimal (0–4), mild (5), moderate (10–14), and severe depression (15–27). The self-report measure has high internal consistency with a Cronbach’s α > 0.80^57^.

#### Gambling Attitudes and Beliefs Survey (GABS)

The GABS^58^ is a 35-item self-report questionnaire assessing dysfunctional attitudes and beliefs about gambling (e.g., illusion of control, gambler’s fallacy). Items have to be answered on a 4-point Likert scale ranging from *strongly agree* to *strongly disagree*. The GABS shows high internal consistency (Cronbach’s α > 0.9^58^. We used a 15-item short form of the GABS with items selected using item response theory^59^ with a total score ranging from 0 to 45. The GABS-15 shows good increment validity^59^.

#### South Oaks Gambling Screen (SOGS)

The SOGS^60^ is the most frequently used self-report measure worldwide in assessing gambling symptoms over the past 6 months. The SOGS is a 20-item questionnaire with sum scores from 0 to 2 indicating no gambling problems, 3 to 4 at-risk gambling, and 5 to 20 pathological gambling. The questionnaire shows good psychometric properties with moderate internal consistency (Cronbach’s α = 0.69) and good convergent validity (*DSM-IV* and *DSM-5*, *r* = .66; Goodie et al.^61^. At post assessment, the timeframe was adjusted to the time since the beginning of the study (i.e., 8 weeks).

#### Web Screening Questionnaire (WSQ)

The WSQ^62^ was used to screen participants for comorbid mental illnesses at baseline (depression, alcohol abuse or addiction, anxiety disorder, obsessive compulsive disorder, and suicidality). The brief web-based screening instrument shows good sensitivity (0.72–1.00) and specificity (0.44–0.77).

#### Subjective appraisal of the program

To measure the acceptance of Restart, participants were asked to answer eight questions about the quality, utility, and applicability of the program. Answers were given on a 4-point rating scale ranging from 1 = *not true at all*, 2 = *somewhat true*, 3 = *mostly true*, and 4 = *completely true*, where ratings from 2 to 4 were counted as positive answers. Moreover, an adapted variant of the German version (Fragebogen zur Messung der Patientenzufriedenheit, ZUF-8)^63^ of the Client Satisfaction Questionnaire (CSQ-8)^64^ was conducted. The original version of the ZUF-8 assesses treatment satisfaction after intensive inpatient treatment. We have adapted the eight items of the questionnaire to ask about satisfaction with the Internet-based intervention. The eight items were scored from 1 (least positive) to 4 (most positive); the exact scale is provided in the results section (Table 5).

#### Baseline characteristics

Several demographic data were assessed at baseline, namely, gender, age in years, high school graduation, nationality, and occupational status. In addition, the following treatment variables were asked about: current psychotherapy status, current psychotropic medication, current use of self-help, age at first gambling, age at frequent gambling, and current gambling suspension status. Furthermore, all participants were asked whether they had received any of the following diagnoses in their past lives: gambling disorder, obsessive–compulsive disorder, anxiety disorder, depression, posttraumatic stress disorder, bipolar disorder/mania, schizophrenia/psychosis, or alcohol/drug dependence. At the end of the baseline assessment, participants were asked whether they had answered all questions truthfully. Those who responded negatively to this question were excluded from the study.

### Statistical analyses

We performed repeated measures ANCOVA ITT sample, CC sample (i.e., considering participants who completed both baseline and post assessment), PP sample (i.e., considering participants who completed both baseline and post assessment and logged into the program at least once), and the frequent user sample (i.e., considering participants who completed both baseline and post assessment and at least two modules). Missing values for ITT analyses were estimated by expectation–maximization algorithm and multiple imputation. Time was used as the within-group factor (pre, post), condition as the between-group factor, and baseline scores as covariate. We used paired sample *t*-tests for analyses of within-group differences. Within-group differences were only analyzed for complete cases. Furthermore, exploratory moderation analysis was performed using the SPSS macro PROCESS^65^ to identify variables with moderating effect on improvement of psychopathology. All analyses were performed using IBM SPSS Statistics 25.

## Data Availability

The data that support the findings of this study are available from the corresponding author upon request.
